# The Theory of Evolution - A Jewish Perspective

**DOI:** 10.5041/RMMJ.10008

**Published:** 2010-07-02

**Authors:** Avraham Steinberg

**Affiliations:** Director, Medical Ethics Unit & Senior Pediatric Neurologist, Shaare Zedek Medical Center, Jerusalem, Israel

**Keywords:** evolution, Judaism, religion and science, fossils, random mutations, big bang

## Abstract

All possible pro and con arguments regarding the theory of evolution have been discussed and debated in the vast literature—scientific, religious, and lay—in the past 150 years. There is usually great zealotry in all debating parties, with mutual intolerance of ideas and concepts, disrespect toward opposing opinions and positions, and usage of very harsh language. This prejudiced approach usually does not allow for a reasonable debate. It is important to look at the facts, assumptions, and beliefs of the theory of evolution in a more calm and humble way.

In this article a comparative analysis is offered between the scientific aspects of the theory of evolution and a Judaic approach to these aspects.

The two sets of human thought—religion and science—are fundamentally different in their aims and purposes, in their methods of operation, in their scope of interest and issues, and in their origin and ramifications. Whenever science surpasses its limits, or religion exceeds its boundaries, it actually is a form of an abuse of both. This has happened to the theory of evolution in a more powerful mode than any other interaction between science and religion.

The agenda of many scientists who promote the theory of evolution is to achieve the goal of understanding the existence of the universe as a random, purposeless, natural development, evolved slowly over billions of years from a common ancestor by way of natural selection, devoid of any supernatural metaphysical power.

Jewish faith perceives the development of the universe in a different way: God created the world, with a purpose known to Him; He established natural laws that govern the world; and He imposed a moral-religious set of requirements upon Man.

The discussion and comparative analysis in this article is based upon the current neo-Darwinian theory, although it seems almost certain that even the new and modern assumptions and speculations will continue to be challenged, changed, and revised as new scientific information will be discovered. The theory of evolution is based upon certain facts, many assumptions, speculations, and interpretations, and some fundamental non-evidence-based beliefs.

Judaism accepts all experimentally proven facts and observations of the theory of evolution. Judaism accepts some of the assumptions and interpretations embedded in the theory of evolution, but rejects other assumptions and speculations which contradict fundamental Jewish beliefs, and which are anyway not scientifically proven; to those Judaism offers different interpretations. Judaism strongly rejects all the extensions of the theory of evolution beyond natural sciences, which endorse the biological assumption of the “survival of the fittest” in commerce and human societies as a whole by justifying claims of social inequality, sexism, racism, Nazism, eugenics, and other moral-social deviations as “laws of nature”.

## INTRODUCTION

Hundreds of books and articles—scientific and lay—have been published on the theory of evolution. There are many differences of opinion among scientists, theologians, and related experts about the facts related to this theory and particularly about their interpretations. In this article I shall present *my* understanding of the relationship between the theory of evolution and Judaism. For the sake of transparency, I hereby state that I am an Orthodox Jew, and hence some of the remarks and observations in this article might be biased by this fact. The bias factor, however, is expressed strongly by all experts debating the theory of evolution, whether scientists, religionists, or creationists. There is great zealotry in all debating parties with mutual intolerance of ideas and concepts, disrespect toward opposing opinions and positions, and usage of very harsh language. This prejudiced approach usually does not allow for a reasonable debate. It is important to look at the facts, assumptions, and beliefs in a more calm and humble way.

## HISTORIC BACKGROUND

Evolutionary concepts and ideas have existed already in ancient times. They were primarily based on philosophical arguments, rather than on empiric data.

The modern theory of evolution based on biological data began to develop in the 18th and 19th centuries by several scientists, mainly by Charles Darwin (1809–1882). Indeed, the modern theory of evolution is most identified with Charles Darwin and his famous book: *The Origin of Species*. It was first published in 1859, and subsequently there six revised editions, the latest one published in 1872.

Among the various explanations and interpretations of the theory of evolution initially offered there were some which did not withstand scientific scrutiny. In addition, various fundamental scientific principles that are today accepted without dispute were rejected for many years by the scientific community because of the “danger” that accepting them might cast doubts on the whole theory. Furthermore, in the early years, various “scientific” theories were offered to strengthen Darwin’s theory, and even scientific forgeries were given to justify the theory of evolution.^1^

Soon after the theory of evolution was established, powerful and hostile struggles ensued between atheistic scientists and the Catholic Church. Scientists at that time fought bitterly against any religious faith, as a reaction against the restrictions that the Church had imposed on scientific thought for many centuries. Darwin’s theory became a prominent component in the agenda of many scientists in denying any religious belief. As a counter-reaction the monotheistic religions developed an antagonistic approach to anything that represented any notion of “evolution”, whether facts or speculations and beliefs. Its espousal was considered to be equivalent to denying one’s religious faith. In the beginning of the 20th century various basic assumptions of the original Darwinian theory were found to be scientifically invalid, and the theory was in disarray. In the 1940s the evolutionists recruited scientists from different disciplines in order to revive and modernize Darwin’s theory. They proposed fundamental changes and developed the modern synthetic theory of evolution, currently known as the neo-Darwinian theory. The discussion in this article is based upon current knowledge and assumptions by scientists, although it seems almost certain that these will continue to be challenged, changed, and revised as new scientific information will be discovered.

## BASIC PRINCIPLES OF SCIENCE AND FAITH

The natural sciences are a continuing effort to discover and increase human knowledge and understanding of the universe based on scientific methods. Using controlled methods, scientists collect observable evidence of natural phenomena, record measurable data relating to the observations, and analyze this information to construct theoretical explanations of how things work. The fundamental aspect of modern science is the experimentally proven data under controlled conditions which confirm or reject the theoretical hypotheses about how phenomena work. It is important to distinguish between conclusions drawn from controlled experiments, and a theory, a speculation, or an assumption. Science has inherent limits, including questions and issues that are beyond scientific scrutiny and ability to answer or even to relate to; neutrality as far as morals and ethics are concerned; and, most importantly, scientific truth is objective, not absolute. Hence, it is constantly altered and changed as new discoveries and facts develop. The mere fact that a scientific theory is accepted by the majority of scientists at a particular time does not prove that it is correct. For example, for many generations it was accepted by all scientists that the world is flat, or that the sun rotates around the earth. At that time, these were regarded as objective truths and accepted scientific facts without doubt or question. Nonetheless, it obviously was wrong. The same applies to all scientific theories, including the theory of evolution, which at first may be widely accepted, but which later may be proven to be partially or totally incorrect. Hence, scientists are required to exercise modesty in debating scientific theories, because history has shown time and again that firmly held scientific theories are rejected, proven wrong, and replaced by other theories. The theory of evolution might prevail and be proven right, but on the other hand its fate might be similar to many previous scientific theories that vanished and were replaced totally or partially by other theories.

Religion is a system of faith and worship, where the believers are totally confident in the truth of the existence of a supernatural divine power. Religion is not based upon experimentally proven facts, but rather on a set of beliefs transmitted historically from generation to generation. To the believers, the religious truth is absolute and unchangeable. Judaism, as a monotheistic religion, places an absolute truth in the existence of an Almighty God, whose very nature is beyond human conception,^2^,^3^ who created the world, established the rules of nature, and commanded a moral-religious practice embodied in the Bible which was given to the Children of Israel on Mount Sinai around 3,300 years ago (around 1290 BC).

The two sets of human thought—religion and science—are fundamentally different in their aims and purposes, in their methods of operation, in their scope of interest and issues, and in their origin and ramifications. Whenever science surpasses its limits, or religion exceeds its boundaries, it actually is a form of an abuse of both. This has happened to the theory of evolution in a more powerful mode than any other interaction between science and religion.

## THE JEWISH FAITH AND THE THEORY OF EVOLUTION

### A) GENERAL AND BASIC PRINCIPLES

The theory of evolution is based upon certain facts, many assumptions, speculations and interpretations, and some fundamental non-evidence-based beliefs. The agenda of many scientists who promote the theory of evolution is to achieve the goal of understanding the existence of the universe as a random, purposeless, natural development, evolved slowly over billions of years from a common ancestor by way of natural selection, devoid of any supernatural metaphysical power.

Jewish faith perceives the development of the universe in a different way: God created the world, with a purpose known to Him; He established natural laws that govern the world; and He imposed a moral-religious set of requirements upon Man. No scientifically proven *facts* negate these statements. Moreover, all experimentally proven facts of the theory of evolution, as well as some of its assumptions and interpretations, are compatible with and accepted by Judaism. Indeed, some authoritative Jewish scholars found no fundamental contradiction between the *factual* parts of the theory of evolution and the Jewish faith, and even view these parts as strengthening the Jewish beliefs as a confirmation of the general scheme of creation.^4^–^8^ The various details in the Biblical story of creation which appear to contradict the scientifically validated portions of the theory of evolution need not be understood literally. Most authoritative Jewish scholars agree that the technical details concerning the creation of the universe, as well as the physical-chemical processes that govern the world, are not necessarily fixed according to the literal wording in the Bible. Hence, there are different opinions and approaches concerning the manner in which God created the universe, the timing of the creation, and His degree of involvement in nature. All these issues can be interpreted in a way compatible with the facts of the theory of evolution. However, scientists who speculate, believe, and interpret the theory of evolution as negating or contradicting the existence of God, God’s creation of the world and Man, or His establishing the rules of nature—all beyond the scope of science and not experimentally proven—deny the fundamental tenets of Judaism, since it is a cardinal axiom of Judaism that God created the world from nothing.^9^,^10^ Many scientists nowadays admit that the theory of evolution is incomplete, and the unanswered scientific questions are numerous. In particular, the theory does not and *cannot* explain the very beginning of the world and the development of life from initial organic material. It cannot, therefore, be considered scientific evidence to refute faith and belief in the creation of the world and of Man by God. Evolution is only a theory; therefore, one can accept that which is fact and experimentally proven and reject that which is an unsubstantiated hypothesis, or replace it by an alternative explanation. Moreover, there is a difference between the *biological* theory of evolution, which portrays the natural evolution, and the extrapolation of this theory to the spheres of beliefs, human behavior, values, and ethics. Some scientists have expanded the biological theory of evolution into a type of a “religion”, explaining the universe and the psycho-ethical and political behavior of Man on the basis of beliefs and speculations which are not experimentally proven and indeed cannot be proven by scientific methods, and hence are beyond the scope of science. There is partial and inconclusive scientific support only for the biologic theory of evolution, but none for the ethical and sociological derivatives of the theory.

### B) SPECIFIC DETAILS OF THE THEORY OF EVOLUTION AND A JEWISH APPROACH

Judaism accepts all experimentally proven *facts and observations* of the theory of evolution. This is based upon the fundamental Jewish concept—stated already by one of the most prominent Jewish theologians, Rabbi Yehuda Ha’levi (1075(?)–1140(?))^11^—that there is not and cannot be a contradiction between scientific knowledge based on controlled experiments and the Jewish religion. It follows that all experimentally proven observations and controlled experiments embedded in the theory of evolution ought to be accepted by Judaism:

- Living creatures differ from generation to generation and have genetic offspring with new and different characteristics because of *random mutations*.
  Nothing in Jewish faith negates the explanation of random mutations as the cause for *intra*-species changes. The micro-evolutionary processes have been demonstrated time and again and hence are experimentally proven facts acceptable by Judaism. Indeed, already early rabbinic authorities described numerous intra-species changes between the Talmudic period and their own. They called it “Nature has changed”. A summary of such changes can be found elsewhere.^12^ There is, thus, a mutual agreement that changes (i.e. mutations) are constantly occurring in nature. There are differences of opinion, however, as to the processes responsible for these changes. All evolution constitutes change, but not all changes constitute evolution. Therefore, one can accept the concept that there are constant changes in nature and in living creatures, but this does not necessarily lead one to accept the full theory of evolution. For example, a table represents a significant change from the pieces of wood from which it was fashioned. Nonetheless, one would not claim that the table spontaneously evolved from the wood. A change occurred, but spontaneous evolution did not. The difference between evolutionists and religionists in interpreting these observations will be discussed later.
- Various *fossils* have been discovered in different geological layers, which point to the existence of different species over time. In general, the earliest creatures, which are also the simplest in structure, are found in the oldest geological layers.
  Nothing in the Jewish faith negates this observation. In fact, the Biblical story of creation in Genesis describes a gradual *creation* of creatures from simple to more complex, and finally to Man. The difference in the interpretation of these observations between evolutionists and religionists will be discussed later.
- Throughout the long existence of the earth several *major catastrophes* occurred due to extreme and abrupt climate changes and meteorite collisions, causing the *extinction* of various forms of life.
  The Bible relates to several such catastrophes, i.e. the Deluge. Also, we find in ancient Jewish sources descriptions of various extinct creatures: the huge sea monsters (*taninim*),^13^ which some interpreters refer to as dinosaurs;^14^ the *tachash*,^15^,^16^ an animal which existed at the time of Moses but is now extinct;^17^ the wild *adanim*,^18^ which some identify as animals resembling humans, such as apes and gorillas;^19^,^20^ the *achbar*, a type of rodent, half flesh and half earth;^21^,^22^ the *salamandra*, which is interpreted to be a type of a lizard;^23^ and others. Moreover, the first-created human being, Adam, underwent significant changes: he was a giant, but when he sinned God placed His hand upon him and diminished him;^24^,^25^ according to one view, Adam was created by God with two countenances and a tail from which Eve was created;^26^,^27^ another view states that he was an hermaphrodite^28^ and the fingers of his hands were fused; only from the era of Noah were human beings born with separated fingers.^29^ In the mystical literature, several types of Man other than Adam are described.^30^ The difference in the interpretation of these observations between evolutionists and religionists will be discussed later.
- Judaism accepts some of the *assumptions and interpretations* embedded in the theory of evolution, but rejects other assumptions and speculations which contradict fundamental Jewish beliefs and which are anyway not scientifically proven:
- One of the strongest pieces of evidence to support the theory of evolution is the discovery of *fossils*. The assumption is that over a long period of time more complex creatures have developed from simpler ones, since in general the earliest creatures, which are also the simplest in structure, are found in the oldest geological layers.
  Judaism rejects the notion that totally new species are developed from lower species. This assumption has never been scientifically proven. Many speculations, inconsistencies, and even falsi-fications surround the story of the fossils. Here are some serious challenges concerning the support of fossils of the theory of evolution: 1) there are major scientific discrepancies about the nature of some fossils or their age; 2) many fossils are recreated on the basis of minimal information, such as fragments of fingers, or a jaw-bone, or a skeleton, or feathers—indeed, there are proofs that significant mistakes occurred in identifying and in interpreting some important findings of fossils;^31^,^32^ 3) although in general more primitive species are found in older geological layers, many observations contradict this fact, i.e. younger fossils were found in deeper geological layers and vice versa; and 4) most importantly, even if it is generally true that more primitive fossils are found in deeper geological layers there is a missing link of the most important evidence, namely the transition of a lower species into a higher one. No one has ever demonstrated a transition from one species to a totally different other species, such as a half fish/half reptile or a half ape/half human as a sign of transition from one species to another. In fact, creatures have always appeared suddenly and fully formed in the fossil record. In the words of Professor David Pilbeam of Harvard University: “our theories have often said far more about the theorists than they have about what actually happened … Virtually all our theories about human origins were relatively unconstrained by fossil data … Many evolutionary schemes were in fact dominated by theoretical assumptions that were largely divorced from data derived from fossils”.^31^
- Genetic changes remain if they result in strong, adaptive, and beneficial characteristics; the process of *natural selection* favors genes that improve capacity for survival and reproduction.
  These assumptions are not necessarily negated by Judaism. However, the whole theory of natural selection and the survival of the fittest has many loop-holes and queries. It is not clear what are considered definite survival advantages or vice versa. Also, the theory of the survival of the fittest is seriously challenged by the fact that the end result of a meaningful “positive” change requires a combination of hundreds of mutations in different locations and directions, over a very long period of time. This theory requires that there should be thousands of generations to develop a new organ such that it can help in the fight for survival. Until it reaches a certain stage of development, the organ is usually an impediment. How, then, could the natural selection process determine what mutations are better during the complex, slow, and lengthy process? And how could this process overcome the many generations of defective offspring until the final “positive” outcome is completed?
- Many species have been *extinct* gradually due to the processes of spontaneous, random natural selection, where the fittest survive and adapt and the weakest disappear.
  It is a fact that many species were extinct, but the assumption that this phenomenon occurred as a slow and gradual process supporting the evolutionists’ theory of survival of the fittest has not been scientifically validated. In fact, a more plausible explanation is the finding of abrupt mass extinction of species because of sudden catastrophic events. This has been interpreted by evolutionists as a “bad luck” of the particular extinct creatures. “Bad or good luck” are clearly not scientific terminologies, and they represent a major loop-hole in the theory of evolution.
- Basic to the theory of evolution is the assumption that the laws and forces of physics, chemistry, geology, astronomy, and biology known to us today have been the same since the beginning of the world. This is termed the *principle of actuality*.
  Judaism can accept this assumption. However, it has not been proven by scientific methods; in fact, there might even be evidence to the contrary. The big bang theory is an example of enormous, abrupt, and rapid changes at the beginning, so why not assume that there were more similar events in the past? Also, there is historic evidence of major catastrophes in the past, such as an abrupt and extreme change in climate (“the ice period”), or a most powerful collision between a huge meteor and our earth. Hence, “jumps” in the development of the world are better substantiated by such historic facts than the theoretical principle of actuality. The acceptance of such an alternative is destructive to the theory of evolution but not to faith in God.
- The processes of development are *random*, beginning from the random or spontaneous creation of an organic molecule to the random evolution from a common ancestor with “positive” mutations which lead to micro- and macro-evolutionary changes, including the development of Man.
  The assumption that the whole universe, including Man, has been developed randomly is merely a speculation and a belief on behalf of the scientists without any scientifically proven evidence. There is no evidence that random variation can play a role in major evolutionary advances. In fact, there is evidence to the contrary, that randomness *cannot* play such a role. Random mutations that can affect positively a change are quite rare; most mutations are insignificant, and a great number of them are actually detrimental and harmful. No scientific theory of probabilities can accept the premise that the complex and complicated structure of the universe, and particularly of life on earth, occurred randomly.^33^,^34^ Two world-famous astronomers, Sir Fred Hoyle and Chandra Wichramasinghe, calculated the probability of such an event and claimed that the probability of such an occurrence is one in ten-to-the-power-of-forty-thousand (1/10^40,000^). In their words, this number “is sufficient to bury Darwin together with his Theory of Evolution”.^35^ Judaism rejects the speculations and beliefs about a *total* random process. On the other hand, Judaism believes that the natural laws were built into the world by its Creator, the Almighty God. However, once the laws of nature have been installed into the world, some processes continue to occur randomly. Already Maimonides (1138–1204) stated clearly that processes concerning nature and the animal kingdom occur spontaneously and randomly without direct interference of God.^36^–^38^
- One of the most challenging problems of the theory of evolution is the *origin of life*. How did original *organic* chemistry transfer into viable *biochemical* molecules?
  The current scientific speculative consensus is that the complex biochemistry that makes up life came from simpler chemical reactions, but it is unclear how this occurred. There are various speculative proposals to clarify this most important part of evolution, but none has been scientifically validated to date. All the enormous knowledge which has enriched us has clarified only mechanisms and individual processes, not essence. An analogy to this situation is someone who comes from an outer planet who observes an earthly transportation network. One can explain to him that the bus or the train is composed of such and such mechanical parts which in unison control the vehicle and allow it to move. However, this person cannot thereby understand how a specific schedule for the bus or train is worked out: how does the mechanical knowledge help him to understand why it stops at certain places, and how does the bus or train arrive at its destination at a certain time? Hence, purely mechanistic explanations are insufficient to understand the controlling mechanisms. Similarly, although we nowadays know physical, chemical, and genetic mechanisms with scientific precision, this knowledge does not explain the essence of life, unless we believe that there is another force which combines all the basic elements precisely into the form and shape of life which we understand. Moreover, all our current advanced knowledge in different fields of the natural sciences has not been able in any way to explain the spiritual aspects of Man: morality, conscience, intellect, thought, will, and their like.
- The currently prevailing scientific view concerning the *age* of the universe assumes that it started about 13.73 ± 0.12 billion years ago (according to the big bang model), our earth is currently assumed to have been formed 4.5 billion years ago, and life appeared on its surface around 3 billion years ago.
  According to the accepted Jewish calendar the world, including life, has been in existence for 5,770 years. This obviously does not concur with the current scientific calculation. This discrepancy can be reconciled in two ways: either by endorsing the standard calculation of the Jewish calendar, and interpreting the scientific data differently; or by endorsing the scientific calculation and explaining the Jewish calendar differently. Both are possible with no hard evidence to the contrary, either by science or by Judaism. The various *scientific calculations* concerning the ages of the universe, the earth, and life on earth are based upon the assumptions that the universe started from zero and that the laws of nature have never changed. Both assumptions are not experimentally proven, and they are based upon belief and logic only. Moreover, natural extreme disasters clearly occurred throughout the long history of earth. In fact, modern theoretical and experimental astrophysics has demonstrated nuclear and radioactive processes which created major and rapid changes in the physical, chemical, and biologic world, rather than orderly, slow, and gradual changes. Hence, the scientific calculations based on physical-chemical data might be grossly mistaken. Therefore, one could assume that the universe was created with advanced physic-chemical characteristics and/or that the physic-chemical nature known to us today was different at the beginning. On the other hand, from a *Jewish point of view* there are numerous ancient sources that point to the fact that the universe is much older than 5,770 years or that one could find evidence for older worlds. Some ancient sources state that God created earlier worlds and destroyed them.^39^,^40^ Other sources state that before Adam, the first man according to Genesis, there were 974 generations which were destroyed because of their sins.^41^ In addition, the time reckoning during the six days of creation, as described in Genesis, might not have been the same as the time reckoning we know today because *a thousand years in Thy sight are but as yesterday when it is past*,^42^ indicating that “a day” to God is different than that of humans.^43^–^45^ Also, the biblical expression “one day” rather than “first day” is distinguished from the expression of the following days of creation which are enumerated as “second day”, “third day”, etc. This is to emphasize that the first day of creation was inherently different than the rest of creation, because there was complete chaos and there was no light; hence, this “day” is beyond measure of time and space. It might be assumed that it was a long “day” with none of the present-day physical, chemical, biological rules of nature. Already some of the most prominent early rabbinic commentators of the Bible emphasized that Genesis does not teach the order of creation (Rashi 1040–1105),^46^ and that our knowledge and understanding of creation is less than a drop in the ocean (Nachmanides 1194–1270).^47^ There are many hidden explanations of the creation of the world, and we do not know the real truth.^47^ Attesting to the thesis that the verses in Genesis are not to be interpreted literally is the observation that the Talmudic Sages differ in many details about the story of creation: whether the heaven or the earth was created first, or whether they were created simultaneously;^49^ whether light was created first, or whether the world was created first;^50^ whether the world was created in the month of *Tishri* or in *Nissan*,^51^ and many more.
- The currently prevailing scientific view of the *beginning of the universe* is the big bang model. It refers to the idea that the universe has expanded from a primordial hot and dense initial condition at some finite time in the past. According to this cosmological model the entire universe was compressed and compacted in a microscopic “ball”, which at a given moment exploded. The unimaginable colossal energy that was released by the explosion created all the galaxies including our solar system.
  This model, in principle, is compatible with Judaism. All Rabbis agree that the beginning of the universe was a creation from nothing. There is, however, disagreement whether the continuation of creation was something from nothing, or whether things then evolved from the original essence. This essence is called *heyuli*^47^,^52^,^53^ (from the Greek *hyle*, meaning original essence), and the source of other creations stems from this original essence.^53^ This original essence could be interpreted as the big bang. In recent years, with the advancement of modern theories in the fields of theoretical and experimental physics, many books and articles were published, trying to give a new meaning to the first verses of the Book of Genesis.^54^,^55^ Nonetheless, without any evidence associated with the earliest instant of the expansion, the big bang theory, by itself not yet experimentally proven, does not and *cannot* provide any explanation for the initial condition; rather, it describes and explains the general evolution of the universe since that instant. It still does not provide any explanation on the very beginning, namely: What was before the microscopic ball? How did it develop? Why did the big bang happen at that particular moment? It is obvious that the human mind cannot go beyond the very beginning, and the very creation is beyond our known scientific rules of nature. Moreover, the model of the big bang does not support the assumption of the eternity of the world, but rather a creation and a beginning.
- The most disturbing problem of the theory of evolution from a religionist’s point of view is its faulty *extension* to fields unrelated to biology and natural sciences. Various scientists, psychologists, sociologists, philosophers, economists, and politicians have abused the theory of evolution, by using it to prove the non-existence of any metaphysical power, by using the biological assumption of the “survival of the fittest” in commerce and human societies as a whole, and by justifying claims of social inequality, sexism, racism, Nazism, eugenics, and other moral-social deviations as “laws of nature”.
  Not only religionists but also many evolutionists are strongly opposed to such extensions of the principles of natural evolution. The acceptance of the supposition that the creation of Man was accidental without purpose or intent leads one to the conclusion that Man’s creation was without a goal and without a plan. Therefore, it may lead to the conclusion that people have a right to ignore the ethical and moral foundations of humanity. This attitude is the basis for the theory of the stronger races having dominion over lower ones in accordance with the randomness of natural selection and survival of the fittest over the weakest in society. Judaism totally rejects all the extensions of the theory of evolution beyond natural sciences.

## Abbreviations:

ID: intelligent design;
IC: irreducible complexity
